# Heavy Metal-Associated (HMA) Domain-Containing Proteins: Insight into Their Features and Roles in Bread Wheat (*Triticum aestivum* L.)

**DOI:** 10.3390/biology14070818

**Published:** 2025-07-05

**Authors:** Mehak Taneja, Santosh Kumar Upadhyay

**Affiliations:** 1 Department of Botanical and Environmental Sciences, Guru Nanak Dev University, Amritsar 143005, India; tanejamehak2@gmail.com; 2 Department of Botany, Panjab University, Chandigarh 160014, India

**Keywords:** *Triticum aestivum* L., heavy metal-associated, HMA domain, phylogeny, *cis*-regulatory elements, expression analysis

## Abstract

The heavy metal-associated (HMA) domain-harboring proteins constitute critical players involved in the transport of various metal ions in plants, and are associated with development and stress responses. Herein, a total of 243 *TaHMA* genes were identified in the bread wheat genome, each of which had a characteristic molecular profile and a distinct chromosomal localization. The TaHMA proteins were distributed in five clades in phylogeny, which differed with respect to the distribution of the key HMA domain. Sub-cellular localization was variable for the TaHMA proteins. Gene structure analysis yielded similar results when compared with the orthologous counterparts. *Cis*-regulatory element analysis produced a range of promoter elements, suggesting their diverse biological roles. Gene duplication analysis revealed a crucial role played by tandem and segmental duplication events in the expansion of *TaHMA* genes. Synteny analysis highlighted the evolutionary relatedness of *TaHMA* genes with those derived from Arabidopsis and rice. Expression analysis provided crucial information about the role of *TaHMA*s in mediating vital responses in the plant body, including the development of tissues and the regulation of various abiotic stress conditions. Overall, the study provides significant cues and evidence to functionally annotate and characterize the differentially expressed *TaHMA*s in order to validate their role.

## Simple Summary

Unprecedented economic growth has resulted in the booming of industries, which in turn has led to rapidly growing heavy metal pollution. Heavy metals have a detrimental effect on plants and animals through the contamination of air, food, and water resources, thereby posing a major environmental and health concern for people across the world. The heavy metal ATPases (HMAs) participate in the absorption and transportation of the otherwise essential metal ions within the living cell, while sequestering the harmful effects of non-essential heavy metal ions. The present study aimed to identify and annotate the complete set of HMA genes and proteins in a major food crop, such as bread wheat. The results revealed an array of diverse characteristics, such as a wide distribution of the HMAs within the cell, while the evolutionary studies pointed out the relatedness of the identified members with other plant species. Overall, the study revealed the role played by these genes in mediating crucial biological processes, such as plant growth, development, and stress responses.

## 1. Introduction

The peril of heavy metal pollution lingers on the modern industrial world, making it one of the significant problems in the present era. The primary sources of heavy metal pollution include fossil fuel burning, milling, mining, and the use of agrochemicals, which lead to the accumulation of a variety of heavy metals. The toxicity posed by heavy metals causes an enormous threat to the survival of various living organisms, including both plants and animals [1,2,3,4,5]. Speaking of crop plants, metal toxicity makes them amenable to several growth and developmental defects and causes impairments in multiple biochemical and physiological aspects, including photosynthesis, respiration, water relations, and mineral nutrition [3,5]. Moreover, heavy metal contamination of the natural ecosystem eventually results in a significant threat to human health. For instance, the normal functioning of crucial organs, like the liver and kidneys, is hampered due to the inhibitory action of cadmium (Cd) [6].

Certain heavy metals, such as zinc (Zn), copper (Cu), manganese (Mn), cobalt (Co), and nickel (Ni), are essential to plants due to their multitude of beneficial roles, such as providing structural support to proteins, serving as enzyme cofactors, etc. However, other heavy metals, including lead (Pb) and Cd, are non-essential, as they are highly toxic to plants and adversely affect crop productivity [7]. Consequently, plants have to continually fine-tune the homeostasis of essential metals. At the cellular level, metal ions are rendered less harmful through various means, including chelation, transport, and vacuolar sequestration. Moreover, various signaling pathways and defense mechanisms are instigated, leading to the synthesis of stress-related proteins, in response to exposure to heavy metals [8]. Heavy metal-associated proteins or heavy metal ATPases, colloquially also referred to as HMPs or HMAs, participate in the absorption and translocation of heavy metal ions, such as Cu^2+^, Co^2+^, Zn^2+^, Pb^2+^, and Cd^2+^, by combining ATP hydrolysis with metal ion transport across cell membranes. HMA proteins can be broadly divided into two sub-families, including a Cu/Ag-ATPase subfamily and a Zn/Co/Cd/Pb-ATPase subfamily, depending on their evolutionary descent and specificity for the metal substrate [4,9,10]. HMA domain-containing proteins are essential for the spatiotemporal transit and detoxification of heavy metal ions. Presently, the *HMA* gene family has been identified and characterized in various plant species, including Arabidopsis (*Arabidopsis thaliana*) [8], areca palm (*Areca catechu* L.) [5], barley (*Hordeum vulgare* L.) [11], maize (*Zea mays* L.) [12], peanut (*Arachis hypogaea*) [13], poplar (*Populus trichocarpa*) [14], rice (*Oryza sativa* L.) [8], sorghum (*Sorghum bicolor* L.) [12], soybean (*Glycine max* L.) [15], and tomato (*Solanum lycopersicum* L) [10].

Bread wheat (*Triticum aestivum* L.) constitutes a major food crop worldwide and is an important source of nutrition for a significant portion of the world’s population. Furthermore, various environmental stressors, including toxic metals, adversely affect the global wheat output. Consequently, there have been reports exploring *HMA* genes in wheat, but surprisingly, they identified only 27 *HMA* genes in the allohexaploid genome of *T. aestivum* [4,16], which is even less than in diploid plants, including Arabidopsis and rice [8]. The reported TaHMAs have been monitored for various vital characteristics, including their gene and protein structure analyses, sub-cellular localization prediction, and phylogenetic analysis [4,16]. However, such comprehensive information has been documented for merely a minor proportion of the otherwise large pool of HMAs occurring in the sizeable genome of polyploid bread wheat. Therefore, the available scientific literature still lacks an accurate and complete identification of this critical gene family in an important food crop, like *T. aestivum*. Hence, considering the requirement of filling this lacuna, we performed exhaustive genome-wide mining of HMA domain-containing proteins in the allohexaploid genome of bread wheat, followed by their comprehensive characterization and expression analyses across various growth and development stages as well as under the influence of various abiotic and biotic stress conditions.

## 2. Materials and Methods

### 2.1. Identification of the HMA-Domain Containing Proteins (TaHMAs) in T. aestivum

A total of 46 and 55 HMP protein sequences of rice (OsHMP1-46) and *Arabidopsis* (AtHMP 1–55) [8] were employed as queries for the BLASTP search against the whole genome data of *T. aestivum* (IWGSC) available in the Plant Ensembl database [17]. In addition, the Plant Ensembl database was scanned using Pfam; PF00403, Intrpro; IPR006121 and IPR036163, Superfamily; SSF55008, and Prosite; PS50846 and PS01047 for the extraction of HMA domain-containing proteins in bread wheat. Non-redundant TaHMA protein sequences were identified by comparing all of the extracted sequences and further verified for the presence of the HMA domain using the NCBI conserved domain database (CDD) database (https://www.ncbi.nlm.nih.gov/cdd/, accessed on 21 August 2023), SMART server (http://smart.embl-heidelberg.de/, accessed on 21 August 2023) [18,19], InterPro (https://www.ebi.ac.uk/interpro/, accessed on 21 August 2023), and PROSITE (https://prosite.expasy.org/, accessed on 21 August 2023) [20,21]. The identified TaHMA proteins were used for homeologous grouping, as done previously [22,23]. The nomenclature of the candidate *TaHMA* genes was determined following the prescribed rules followed in earlier studies [22,23]).

### 2.2. Elucidation of HMA Characteristic Parameters

The gene locations were recorded from Plant Ensemble (http://plants.ensembl.org/Triticum_aestivum/, accessed on 13 November 2023) and confirmed through the BLASTN search against the chromosomal sequences of *T. aestivum* procurable through URGI (https://urgi.versailles.inra.fr/blast, accessed on 13 November 2023). The information retrieved was illustrated using PhenoGram (http://visualization.ritchielab.org/phenograms/plot, accessed on 13 November 2023) in order to pictorially depict the location of the gene IDs on their respective chromosomes [24]. Various protein characteristics, such as peptide length, length and nature of the DNA strand, molecular weight (kDa), and isoelectric point (pI), were also retrieved using the Plant Ensemble tool so as to comprehensively annotate each of the TaHMAs identified in the present work.

### 2.3. Gene Structure Identification and Cis-Regulatory Element Analysis in TaHMAs

The composition and position of exons/introns and intron phase distribution in *TaHMA* genes were analyzed bioinformatically by aligning the coding and genomic sequences using the gene structure display server (GSDS 2.0) [25] (http://gsds.gao-lab.org/, accessed on 20 October 2024). Next, to analyze the *cis*-regulatory elements in the *TaHMA* gene family, the region about 1.5 kb upstream from the start codon (ATG) of the gene was selected manually and submitted to the PlantCare database [26] in order to analyze the *cis*-acting regulatory elements in the promoter regions. The results were visualized by using TBtools software v2.154 [27].

### 2.4. Multiple Sequence Alignment and Phylogenetic Analysis

The full-length HMA protein sequences of *T. aestivum*, *O. sativa*, and *A. thaliana* were used for the evaluation of evolutionary relationships. Thereafter, two separate trees were used for analysis, of which, one exclusively had HMAs extracted from *T. aestivum*, while the other consisted of HMAs derived from the three aforementioned plant species. Multiple sequence alignments were performed using ClustalW 2.1 [28] and subsequently uploaded to MEGA7.0 for construction of the phylogenetic trees according to the maximum likelihood method with 1000 bootstraps [29]. The resultant tree files were uploaded to the iTOL online tool (http://itol.embl.de/index.shtml, accessed on 20 March 2025) in order to achieve visual enhancement [30].

### 2.5. Domain Architecture, Sub-Cellular Localization, and Motif Analyses of TaHMAs

As described previously, the CDD and SMART servers were used for the distribution analysis of HMA conserved protein domains in the resultant TaHMA protein sequences. The ensuing data for certain selected TaHMA proteins based on their clade distribution was given as an input file to the IBS server (https://ibs.renlab.org/, accessed on 13 March 2025) to obtain the resultant domain diagrams [31]. The sub-cellular localization of HMAs was predicted using the WoLF PSORT (https://wolfpsort.hgc.jp/, accessed on 14 November 2023) protein localization predictor [32]. The resultant cellular signal values were plotted using Microsoft Excel. Conserved motif analysis was performed by the MEME Suite version 5.0.5 (https://meme-suite.org/meme/tools/meme, accessed on 8 December 2024), which is an online software for the prediction of conserved motifs [33]. The distribution type of motifs was set to zero or one occurrence per sequence (zoops). The number of motifs and the minimum and maximum widths were set as 10, 6, and 50, respectively.

### 2.6. Identification of Duplicated Gene Pairs, Calculation for Ks/Ka, and Synteny Analysis

The desirable *TaHMA* gene pairs fulfilling the criteria for segmental gene duplication, i.e., showing a percentage similarity of ≥80%, were manually selected from the percentage identity matrix prepared using the MAFFT server. Furthermore, two or more genes residing within the chromosome range of 200 kb were categorized as tandem gene duplication events. The Ka/Ks values were calculated using the TBTool employing the CDS sequences, protein sequences and gene duplication pair as input files. The divergence time (T) for each duplicated gene pair was calculated using the method and/or formula according to which T = Ks/2λ MYA, where λ = 6.5 × 10^−9^ and MYA = 10^−6^ [4,22]. Additionally, synteny analysis was conducted to illustrate the evolutionary relatedness between wheat, rice, and Arabidopsis HMA proteins, wherein protein sequences for all of the retrieved HMA proteins were fed as input files to a web-based sequence relationship viewer tool, Circoletto (https://tools.bat.infspire.org/circoletto/, accessed on 8 March 2025), using the default parameters [34].

### 2.7. Expression Profiling

Expression analysis of *TaHMA* genes was carried out in different tissue developmental stages and under biotic and abiotic stress conditions, employing high-throughput RNA-seq data (wheaturgi.versailles.inra.fr/files/RNASeqWheat/) available for community access on the NCBI and URGI databases [35]. The variation in expression across the various tissue developmental stages was analyzed amongst the three developmental stages for each root, leaf, stem, spike, and grain in duplicate. The FPKM (fragments per kilobase of transcripts per million mapped reads) values were calculated as per the RNA-seq data [36]. Selective *TaHMA* genes that presented an FPKM of >10 in one or more tissue developmental stages were retained and used for heat map construction. Furthermore, the effect of heat–drought treatment was studied with the usage of high-throughput RNA-seq data retrieved in duplicate upon 1h and 6h of heat stress (HS; 40 °C), drought stress (DS; 20% polyethylene glycol), and combined heat and drought stress treatment (HD) [37]. To analyze the influence of salt stress, RNA-seq data generated at 6 h, 12 h, 24 h, and 48 h post-150 mM NaCl treatment were utilized to analyze the differential expression of the *TaHMA* genes in the present study [38]. The expression readings were evaluated by calculating the fold change under various stressors in comparison to their controls, and *TaHMA* genes showing 2-fold up- or downregulation were considered differentially expressed. The Hierarchical Clustering Explorer 3.5 (http://www.cs.umd.edu/hcil/hce/, accessed on 4 March 2025) was utilized to construct heat maps throughout all of the expression profiling studies [39].

## 3. Results

### 3.1. Identification, Chromosomal Distribution, and Localisation of TaHMAs

The extensive BLAST search against the wheat genome in the Plant Ensembl database resulted in the discovery of 243 non-redundant protein sequences. For the sake of maintaining uniformity and avoiding any ambiguity, the resultant genes identified in the bread wheat genome for heavy metal transport were named *HMA*s in the present study. It is imperative to mention the methods employed in the previously published reports where HMA homologs from *Brachypodium distachyon* and *A. thaliana*, i.e., *BdHMA1-9* and *AtHMA1-8.2*, respectively, were used for BLAST searches to identify the *HMA*s in the wheat genome [4,16]. This is in stark contrast with the present investigation, where a more rigorous approach was applied to identify all of the candidate *TaHMA* genes. Furthermore, it would also be worthy here to make note of the genes that have already been been annotated as *TaHMA*s [4] and that were also obtained in the present analyses, including (as per the nomenclature prescribed for the *HMA* genes identified in the present study) *TaHMA23-A*, *B*, and *D*, *TaHMA53-A* and *D*, *TaHMA71-A*, *B*, and *D*, *TaHMA78-A*, *B*, and *D*, *TaHMA79-A*, *B*, and *D*, *TaHMA84-A*, *B*, and *D*, *TaHMA88-A*, *B*, and *D*, and *TaHMA91-A*, *B*, and *D* (Table S1).

**Figure 1:**
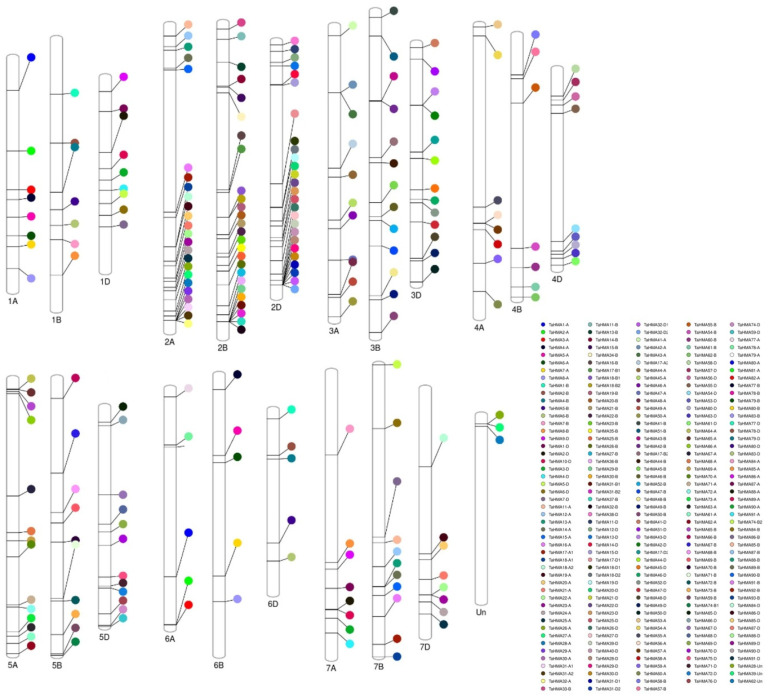
Chromosomal distribution of *TaHMA* genes on chromosomes 1–7 derived from each of the A, B, and D sub-genomes of *T. aestivum*. The localization of each gene is represented by an explicit color assigned to each gene ID.

Chromosomal localization analysis revealed that the *TaHMA* genes were present on all 7 chromosomes derived from the A, B, and D sub-genomes in bread wheat. In total, a maximum of 76 *TaHMA* genes were contained on chromosome 2, while at least 3 genes were reported to be situated on chromosome Un (Figure 1). Eight *HMA* genes were located on 1A, seven on chromosome 1B, and nine on chromosome 1D. For chromosome 2, 24 genes were localized on chromosome 2A and 26 genes each on chromosomes 2B and 2D. Similarly, for chromosome 3, a total of 11, 13 and 13 genes were distributed on chromosome 3A, 3B, and 3D, respectively. Amongst the chromosomes 4, 5, 6, and 7, the number of *HMA* genes that were located on each of the chromosomes derived from the A, B, and D sub-genomes equaled to 8, 7, and 9 for chromosomes 4A, 4B, and 4D; 13, 11, and 12 for chromosomes 5A, 5B, and 5D; 6, 5 and 5 for chromosomes 6A, 6B, and 6D; and 8, 11, and 8 for chromosomes 7A, 7B, and 7D, respectively.

### 3.2. Physiochemical Properties and Sub-Cellular Localization Analyses of TaHMAs

A range of characteristic properties, including physicochemical parameters, such as the molecular weight, length of the peptide chain, isoelectric point, etc., composition and arrangement of domains, occurrence of exon/intron in *TaHMA* genes, and motif analysis, employing the amino acid sequences for the entire set of TaHMA proteins were analyzed through various bioinformatic tools and databases, the results of which are discussed in the subsequent sections. Speaking of the length of the peptide chain, TaHMA47 group proteins (TraesCS3A02G347400; TraesCS3B02G379100; TraesCS3D02G341000) contained only 72 amino acids (aa), whereas a maximum of 1564 aa were present in TaHMA33-B (TraesCS2B02G033500). The molecular weight of the TaHMA proteins ranged from 175.391 kDa for TaHMA33-B to 8.09 kDa for TaHMA47-A. In case of the isoelectric point (pI), TaHMA17-B2 had the highest pI value of 11.86, while TaHMA90-B had the minimum pI, equaling 4.22. Furthermore, of all the total *HMA* genes identified in *T. aestivum*, 132 were located on the forward strand while the remaining 111 were predicted to be on the reverse strand (Table S1).

Sub-cellular localization predicted through the WOLF-PSORT tool provided a range of signal values, which helped in determining the reliability of the prediction for each of the protein sequence given as input to the server. Diverse sub-cellular sites were predicted through in silico analysis for the various TaHMA proteins under study, including the cytoplasm, which showed a positive signal value (i.e., ≠0) for 182 TaHMA proteins, followed by the chloroplast, for which 167 TaHMA proteins generated a putative signal. Furthermore, another popular site of localization that was predicted for the TaHMA proteins was the nucleus, where 152 TaHMAs procured a signal value. The mitochondria and the plasma membrane were sites of localization for 115 and 86 TaHMA proteins, respectively (Figure S1). Furthermore, the majority of homoeologous TaHMAs derived from each of the A, B, and D sub-genomes in bread wheat were predicted to have similar cell localization; for instance, TaHMA79-A, TaHMA79-B, and TaHMA79-D were predicted to be localized to the plasma membrane, the vacuole, and the endoplasmic reticulum with approximately similar signal values for each of these sub-cellular compartments, respectively (Figure S1).

### 3.3. Gene Structure Analysis

**Figure 2:**
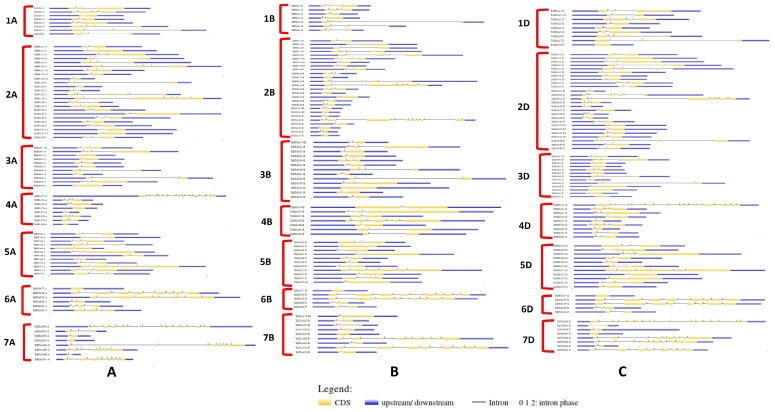
Illustration of the gene structure composition for various *TaHMA* genes in a sub-genome and chromosome-wise manner. (**A**)—*TaHMA* genes derived from the A sub-genome; (**B**)—*TaHMA* genes derived from the B sub-genome; (**C**)—*TaHMA* genes derived from the D sub-genome.

Gene structure analysis was done by aligning the coding sequences with the genomic sequences using the GSDS server 2.0 The results of gene structure display were analyzed sub-genome-wise since the tool was unable to process the sequence data for the 243 *TaHMA* genes in a single run (Figure 2A–C). The presence of introns disrupted the coding sequences of the majority of the *TaHMA* genes. Ten *TaHMA* genes, including *TaHMA9-D*; *TaHMA50-A*, *TaHMA50-B*, *TaHMA50-D*, *TaHMA77-A*, *TaHMA77-B*, *TaHMA77-D*, and *TaHMA87-A*, *TaHMA87-B*, *TaHMA87-D*, were without introns. The rest of the *TaHMA* genes harbored one or multiple introns. A total of 56 *TaHMA* genes possessed 1 intron, 126 possessed 2 introns, 45 possessed 3 introns, 6 possessed 4 introns, and 9 possessed 5 introns. Only one group of genes, i.e., *TaHMA79-A*, *B*, and D harbored 7 introns. In total, 11 *TaHMA* genes possessed 8 introns, whereas 9, 12, and 15 introns were harbored by *TaHMA33-B*, *TaHMA78-B*, and *TaHMA84-A*, respectively. Furthermore, 3 genes possessed 16 introns. The highest number of introns was present in *TaHMA53-A*, corresponding to 17 introns. Lastly, intron phase analysis using the gene structure diagram for the *TaHMA* genes revealed that ~48%, ~46%, and ~6% of introns occurred in phases 0, 1, and 2, respectively.

### 3.4. Cis-Regulatory Element Analysis

**Figure 3:**
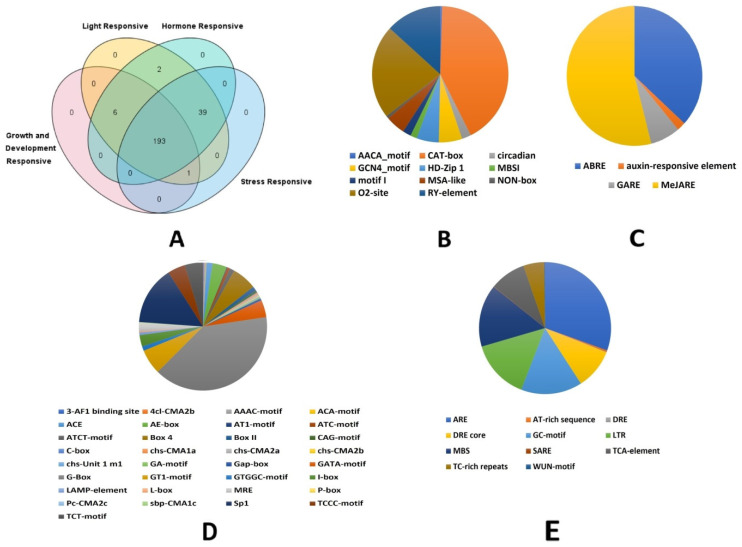
*Cis*-regulatory elements retrieved from the 1500 bp upstream region of the *TaHMA* genes. (**A**)—Venn diagram showing the category-wise prevalence of the *cis*-elements with 193 *TaHMA* genes harboring the entire category list. (**B**–**E**)—Pie charts showing the relative proportion of various elements recorded for growth and development, hormone responsiveness, light-sensitive, and stress-specific elements, respectively.

The analysis of the 1500 bp upstream region of the coding sequences for each of the 243 *TaHMA* genes revealed an array of *cis*-acting regulatory elements involved in diverse biological phenomena ranging from growth and development, light response, and hormone-specific and stress responsiveness (Figure S2). Furthermore, to assess the distribution of various promoter elements amongst the entire set of *TaHMA* genes identified in the present study, a Venn diagram was constructed (Figure 3A). By determining the gene list specific for each function and/or broad category identified in the present study, we identified a total of 193 common gene IDs, which contained the *cis*-acting elements from each of the 4 broad categories. In contrast, 39 genes (e.g., *TaHMA1-A* and *D*, *TaHMA6-A*, *B*, and *D*, *TaHMA22-A* and *D*, *TaHMA23-A*, *TaHMA26-D*) mutualistically consisted of various light-, hormone-, and stress-responsive elements. Another, 6 (*TaHMA16-A*, *TaHMA18-B1*, *TaHMA25-A*, *TaHMA41-B*, *TaHMA80-A* and *TaHMA84-A*), and 2 (*TaHMA5-B* and *TaHMA36-B*) *TaHMA* genes were identified that strictly had growth and development-specific and light and hormone-responsive elements in common, respectively, whereas *TaHMA85-D* was observed to solely harbor the growth and development-specific and light- and stress-responsive *cis*-acting elements. Additionally, pie charts showing the relative proportion of each of the *cis*-regulatory elements identified in the present study were also sketched category-wise (Figure 3B–E). For instance, amongst the growth and development-responsive *cis*-acting elements, the CAT-box (*cis*-acting regulatory element related to meristem expression) was found in the highest abundance, followed by the O2-site element (*cis*-acting regulatory element involved in zein metabolism regulation) amongst the eleven types of elements identified in the present study.

### 3.5. Phylogeny Analysis

**Figure 4:**
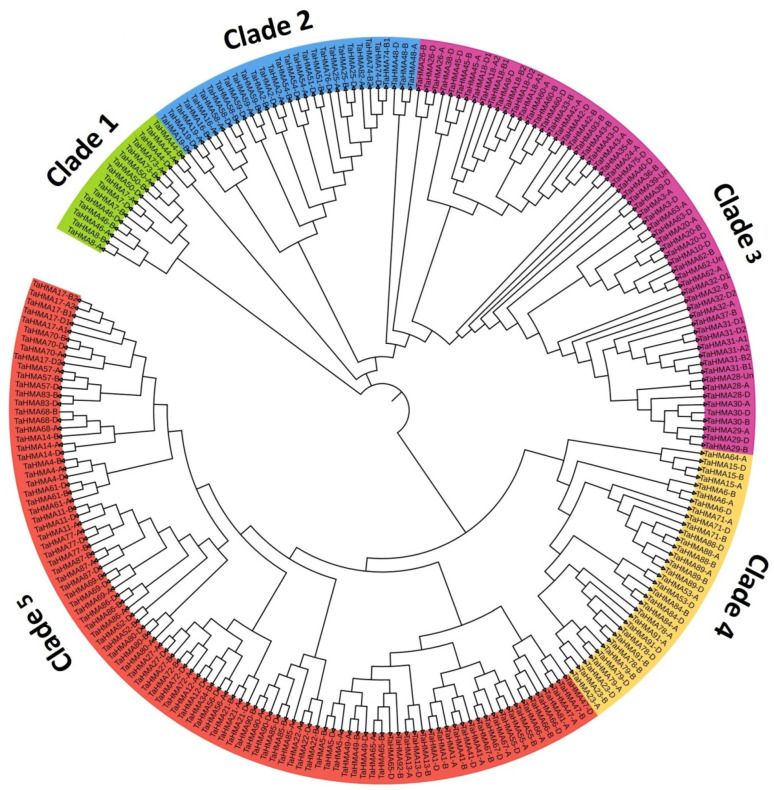
Phylogenetic tree constructed using full-length TaHMA protein sequences showing the evolutionary relationship. Five different clades represented by distinct colors correspond to the clade-wise domain composition as pictorially represented in Figure 5.

**Figure 5:**
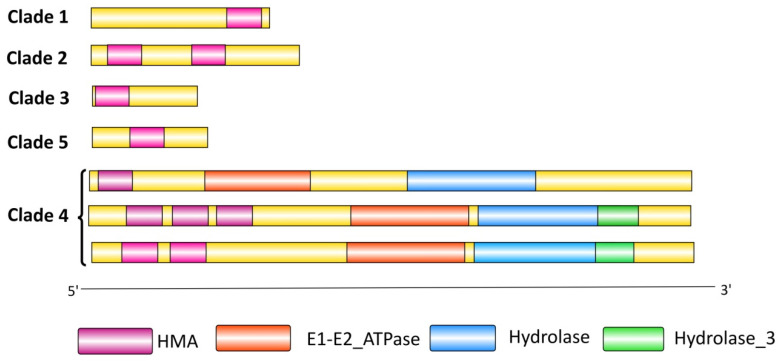
Domain architecture for the TaHMA proteins is represented in a clade-wise manner to depict the major domain architectural styles and/or variations observed for the HMA proteins in the present study.

Phylogenetic analysis conducted to reveal the evolutionary relatedness amongst the 243 TaHMA proteins reported herein revealed five distinct clades, as observed from the circular phylogenetic tree (Figure 4) obtained using the full-length amino acid sequences of HMA proteins of *T. aestivum*. Clade 1 consisted of the TaHMA proteins harboring the HMA domain at the C-terminus, while clade 3 consisted of the TaHMA proteins with the HMA domain located at the N-N-terminus. Furthermore, clade 2 was composed of the majority of TaHMA proteins, containing two HMA domains, unlike clade 5, where the TaHMA proteins clustered together primarily consisted of a single HMA domain. Clade 4 was comprised of the TaHMAs harboring the characteristic domains of HMAs in addition to the HMA domain, which was either occurring singly, twice, or in some instances, thrice in a peptide sequence (Figure 4 and Figure 5). Moreover, the amino acid sequences of rice and Arabidopsis HMPs were employed in order to analyze the extent of evolutionary association of TaHMAs reported in the present study with those derived from the model plant species. A total of 55, 46, and 243 full-length protein sequences derived from *A. thaliana*, *O. sativa*, and *T. aestivum*, respectively, were employed for the construction of an additional phylogenetic tree using similar computing parameters to assess the evolutionary relationship of TaHMAs with their orthologous counterparts (Figure S3). The clustering of the proteins derived from these three plant species was in agreement with the domain distribution and correlated well with other parameters, including the length of the peptide sequence, as described in the Section 4.

### 3.6. Domain Composition and Distribution Analysis

Domain composition analysis revealed majorly occurring domains for the TaHMA protein sequences, including HMA (pfam00403); E1-E2_ATPase (pfam00122); Hydrolase (pfam00702); and Hydrolase_3 (pfam08282). Five characteristic domain patterns were identified for all of the 243 TaHMAs, which coincided with the clade-wise distribution of the TaHMA proteins. For clades 1, 3, and 5, the majority of the TaHMA proteins belonging to each of these groups consisted of a single HMA domain, which in turn varied with respect to its location within the protein, i.e., either at the N or C terminus or somewhere within the two terminations. The rest of the TaHMAs fitted in clades 2 and 4 with the occurrence of one or more HMA domains, i.e., two and/or three in association with the characteristic domains for the HMAs, including E1-E2_ATPase, Hydrolase, and Hydrolase_3 (Figure 5). There were instances where we could not verify the occurrence of the HMA domain for certain TaHMA proteins through these popularly used domain servers. However, the ensemble database verified their identity as the proteins belonging to the HMA superfamily.

### 3.7. Analyses of Motif Diversity

MEME is a powerful tool for discovering novel, recurring fixed-length motifs in the input sequences; therefore, this tool was employed in the present study to elucidate ‘zero or one occurrence per sequence’ of motif sites in the TaHMA protein sequences. The input settings resulted in the discovery of ten motifs. Owing to the huge sequence load, the distribution of motifs in the AA sequences was studied sub-genome-wise and, therefore, was presented in this study accordingly. A visual inspection of the motif distribution revealed the predominance of certain motifs, including motif 1, motif 2, and motif 4, whereas certain motifs, such as motifs 1 and 7, were found occurring in close association with each other, which might point towards their putative role in mediating a common function (Figure S4). Furthermore, clade-wise speaking, motifs 1 and 2, along with motif 7 in some instances, were localized at the C terminus for the members of clade 1 (e.g., TaHMA7-A, B, and D; TaHMA44-A, B, and D), while a similar set of motifs, i.e., 1, 2, and 7, occurred twice along the length of the peptide sequence (e.g., TaHMA16-A, B, and D) for clade 2, thereby suggesting that the distribution of motifs was coherently related to the domain distribution for the TaHMAs in the current study. In a quest to further establish the biological significance of the various motifs identified by MEME, it was observed that motifs 1, 2, and 7 putatively belonged to the HMA domain. Moreover, motifs 3, 6, 8, and 9, along with motifs 5 and 10, could be mapped to the E1-E2_ATPase domain and the hydrolase domain, respectively, due to their predominance in the members of clade 4 (Figure 4, Figure 5 and Figure S4).

### 3.8. Gene Duplication Events and Ka/Ks Analyses

**Figure 6:**
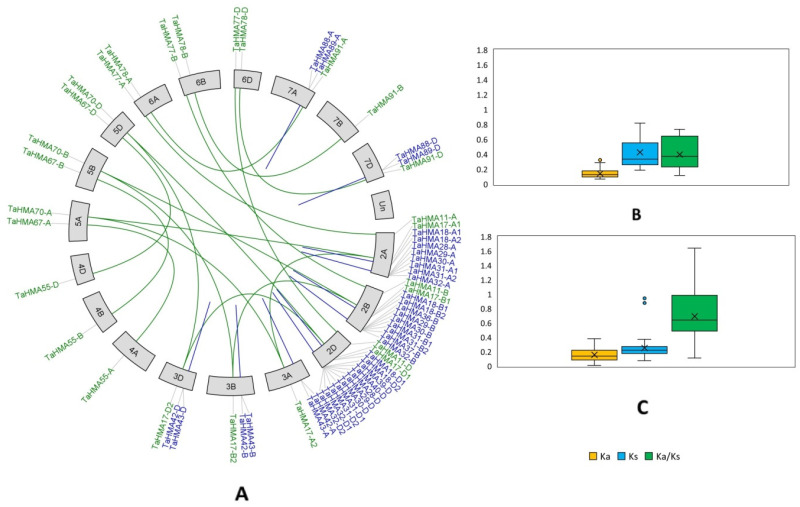
Gene duplication analysis for the *TaHMA* genes. (**A**)—Segmentally and tandemly duplicated gene pairs plotted using TBtools-II v2.154 in a Circos fashion represented by the green- and blue-colored lines, respectively. (**B**,**C**)—Box-and-whisker plots showing the distribution of Ka (orange), Ks (blue) and Ka/Ks (green) values obtained for the segmentally and tandemly duplicated gene pairs, respectively. Each box (encompassing whiskers) shows the median (central line), mean (cross on the box plot) and outliers. The Y-axis contains the values obtained for the Ka, Ks, and Ka/Ks.

In the current study, a total of 18 pairs of segmental and 43 pairs of tandemly duplicated genes were revealed. For instance, *TaHMA17-A1*, *B1*, and *D1* of chromosome 2 formed a segmentally duplicated gene pair with *TaHMA70-A*, *B*, and *D*, respectively, derived from chromosome 5, while *TaHMA28-A* and *TaHMA29-A* constituted a tandemly duplicated gene pair localized on chromosome 2A (Figure 6A). In order to evaluate the selection pressure on the duplicated *TaHMA* gene pairs, Ka or the nonsynonymous substitution rate, Ks or the synonymous substitution rate and eventually, Ka/Ks or the nonsynonymous to synonymous substitution ratio values were evaluated for all of the duplicated *TaHMA* gene pairs reported in the present study. The value of Ka/Ks for the tandemly duplicated gene pairs ranged from 1.642 to 0.112, with an average value of 0.692. For the segmentally duplicated gene pairs, it ranged from 0.735 to 0.118, with an average value of 0.398 (Figure 6B). Furthermore, we reported average divergence time values of ∼19 MYA and ∼33 MYA for the tandemly and segmentally duplicated *TaHMA* gene pairs under study, respectively (Table S2).

### 3.9. Synteny Analysis

**Figure 7:**
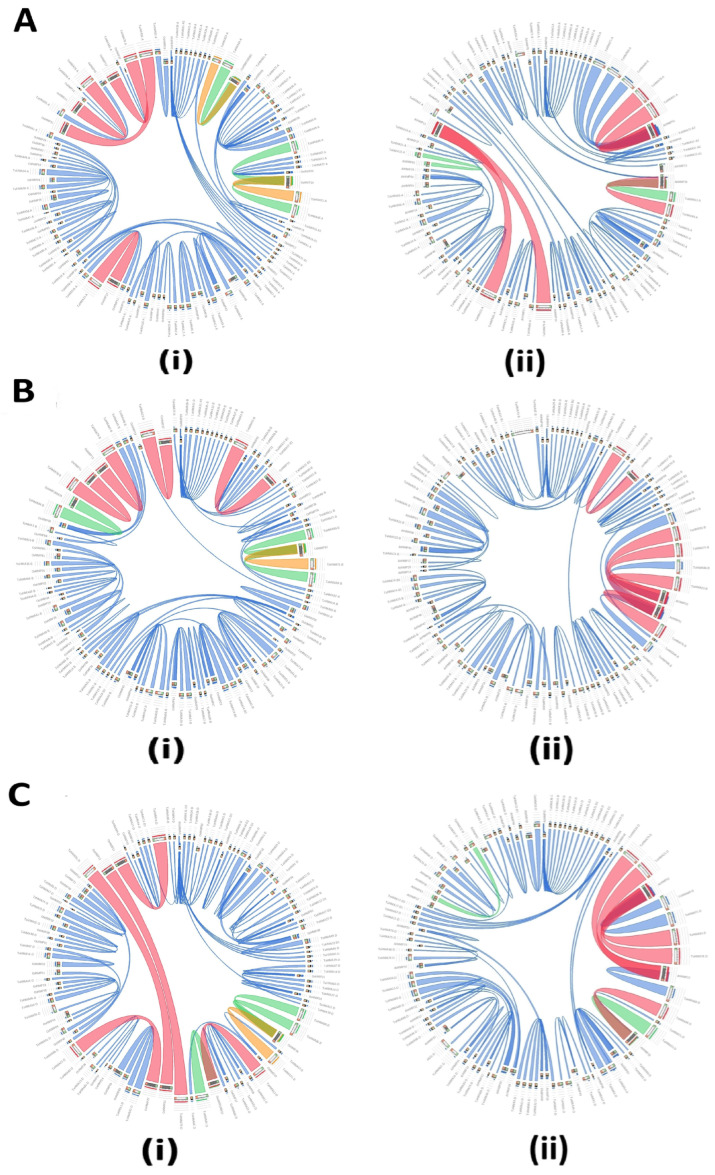
Circos-style plots obtained using the Circoletto tool to analyze the orthologous and/or syntenic relationship between the TaHMAs, OsHMAs, and AtHMAs. (**Ai**,**ii**–**Ci**,**ii**)—Syntenic plots constructed using the full-length amino acid sequences of AtHMAs and OsHMAs, with the TaHMA proteins specific to the A, B, and D sub-genomes, respectively.

A synteny analysis conducted to assess the evolutionary relationship of wheat *HMA* genes with those of Arabidopsis and rice also revealed interesting results. Inside the circle, ribbons correspond to the local alignments BLAST produced in four varying colors corresponding to blue, green, orange, and red, which represent the four quartiles up to the maximum score of 80% shown by red, whereas the one with 20% of the maximum score is colored blue [34]. In the present study, several *TaHMA* genes were shown to have sequence similarities of varying degrees with one or the other *AtHMA* and/or *OsHMA* genes, as illustrated in the Circos diagrams (Figure 7). Moreover, synteny analysis has been widely employed as a tool to infer the putative orthologous genes [40]. For instance, *TaHMA91-A*, *B*, and *D* genes were orthologous to the *OsHMP33* gene. *TaHMA91* group genes also had a syntenic relationship with *AtHMP51*. The latter was orthologous to various other *TaHMA* genes, including *TaHMA71*, *TaHMA78*, *TaHMA88*, and *TaHMA89* group genes (Figure 7(Ai,ii–Ci,ii)).

### 3.10. Expression Analyses

**Figure 8:**
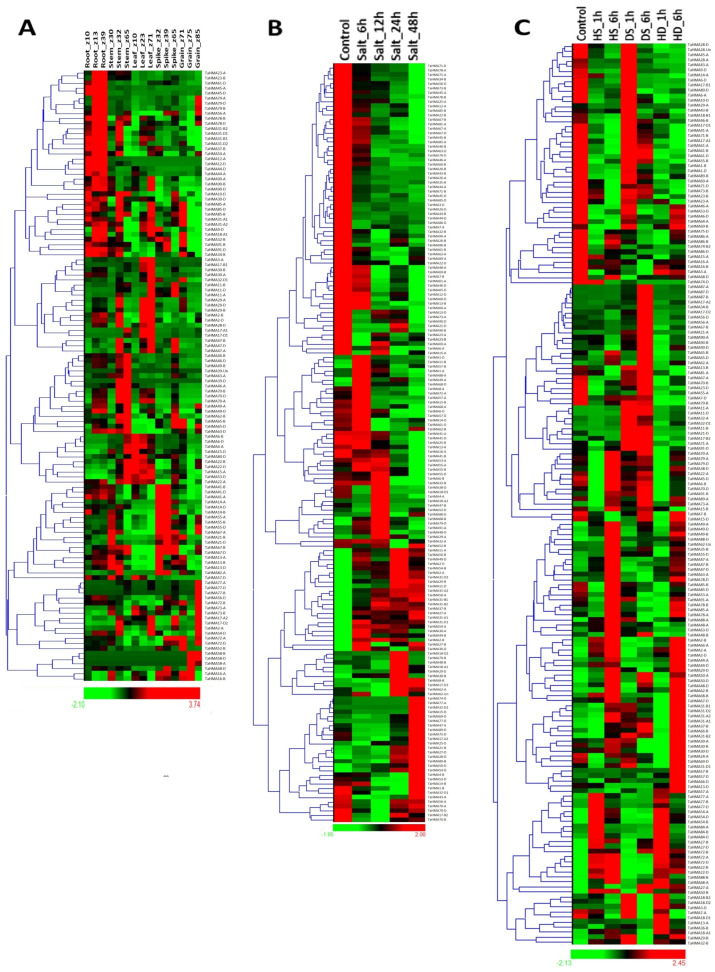
Heat map expression plots constructed using the RNA-seq data intended for various tissue developmental stages represented in the Zadoks scale (**A**), and under salt stress (**B**) and heat, drought, and their combined action (**C**) at various time points for the *TaHMA* genes under study.

RNA-seq data revealed that various *TaHMA* genes were expressed in one or multiple tissues of wheat, and a particularly higher expression was observed in various developmental stages of the root for the majority of the *TaHMA* genes, as evident from the heat map (Figure 8A–C). For instance, *TaHMA90* (*TaHMA90-A*, *B*, and *D*) and *TaHMA85* (*TaHMA85-B* and *D*) group genes were consistently highly expressed in all three stages of root development, i.e., root_z10, root_z13, and root_z39, under study. In various developmental stages of the stem, *TaHMA6* group genes, i.e., *TaHMA6-A*, *B*, and *D*, exhibited significantly high expression during the later stages, like the stem_z65 stage, while *TaHMA11* (*-A*, *-B*, and *-D*) and *TaHMA16* (*-A* and *-B*) had a higher FPKM during the initial stages of stem development, i.e., stem_z30 and stem_z32. Speaking of the developmental stages of the leaf, *TaHMA6-A*, *B*, and *D* had an evidently higher expression during the initial leaf developmental stages, leaf_z10 and leaf_z23. On the contrary, *TaHMA11* group genes, i.e., *TaHMA11-A*, *B*, and *D*, had consistently higher expression during the leaf_z71 stage. *TaHMA29-B* and *D* and *TaHMA90-A*, *B*, and *D* highly expressed during one or the other leaf tissue developmental stage(s), as is evident from their FPKM values. For the various developmental stages of the spike, *TaHMA* gene groups, including *TaHMA11-A*, *-B*, and *-D*, possessed noticeably higher expression values for the later developmental stage of the spike, i.e., the spike_z65 stage, while *TaHMA16* and *TaHMA85* group genes, i.e., *TaHMA16-A* and *B* and *TaHMA85-B* and *-D*, had uniformly higher expression values throughout all developmental stages of the spike tissue in wheat. Lastly, in the case of developmental stages of the grain, *TaHMA58-A*, *-B*, and *-D*, *TaHMA72-A*, *-B*, and *-D*, and *TaHMA77-A*, *-B*, and *-D* were highly expressed in one or the other developmental stage(s), encompassing grain development, including grain_z71, grain_z75, and grain_z85 (Figure 8A).

In response to various abiotic stress conditions, including heat, drought, and a combination of heat and drought stress, the various *TaHMA* genes identified showed variable fold changes in their expression values throughout the various stages under study. For instance, *TaHMA77* group genes, i.e., *TaHMA77-A*, *B*, and *D*, were consistently highly upregulated during the initial and later stages of heat stress and the combination of heat and drought stress treatments. In the case of drought stress, *TaHMA21-A* and *TaHMA87-D* exhibited high upregulation in their expression (Figure 8C). In the case of salt stress, which constitutes another significant abiotic stressor, various *TaHMA* genes showed highly upregulated expression in one or several stages of the salt stress treatment(s). Notably, a high fold-change was seen in the expression values of *TaHMA* genes, including *TaHMA18-A1* (∼143 folds) during salt_24 h, *TaHMA29-A* (∼360 folds) during salt_12 h, and *TaHMA32-D2* (1130 folds) during salt_48 h stage (Figure 8B).

## 4. Discussion

A range of scientific literature is available on the genome-wide identification and characterization of *HMA* genes in a plethora of plant species amongst both dicotyledons and monocotyledons [5,8,11,12]. Moreover, sequencing of the genome of bread wheat has revolutionized the novel discovery of a plethora of crucial gene families in this staple cereal crop [41,42,43,44,45,46]. Resultantly, there are reports where the *HMA* gene family has also been previously explored in bread wheat to some extent. In the studies conducted by Zahra et al. [16] and Batool et al. [4], 27 *HMA* genes have been reported in the allohexaploid genome of *T. aestivum*. Nevertheless, in the present study, we performed a deep analysis and identified a total of 243 *TaHMA* genes. Consequently, previous studies conducted on the exploration of *HMA* genes have revealed a minuscule proportion of the total genes accountable for heavy metal transport in *T. aestivum*. Moreover, it was important to characterize these genes and their encoding proteins, employing various in silico tools, which are indispensable for a better understanding of them. Similar approaches for the genome-wide identification and characterization of the HMA gene family has been adopted for other model plant species. In Arabidopsis and wheat, a total of 10 and 27 HMAs, respectively, were reported by Zahra et al. [16]. In the case of *Oryza sativa*, 9 HMA members have been proposed, while 27 and 9 HMAs with conserved domains have been reported in *T. aestivum* and *T. urartu*, respectively [4]. Strikingly, Li et al. [8] reported 46 and 55 candidate *HMP* genes in Arabidopsis and rice, respectively. These differences in the research findings are probably attributed to the distinct search parameters and criteria utilized by the various laboratories to identify the gene pool for any particular crop species, which also explains the difference in the results observed in the present study in comparison to the literature published previously.

Key characteristics of TaHMA proteins were recorded and analyzed for comparison with their orthologous counterparts. A diverse range in the peptide length of the 243 TaHMA proteins observed in the present study coincided well with the previous studies, where the amino acid length for various HMA proteins ranged from 73 to 1302 aa, 428 to 1030 aa, and 626 to 1228 aa in *S. lycopersicum*, *A. catechu*, and *P. trichocarpa*, respectively [5,10,14]. Moreover, the length of various AtHMA and TaHMA proteins documented by Zahra et al. [16] ranged from 542 to 1172 and 262 to 1071 amino acids, respectively. The chromosomal localization of the 27 *TaHMA*s reported previously is restricted to the five out of seven chromosomes across the A, B, and D sub-genomes, including chromosomes 2, 4, 5, 6, and 7, unlike the present study, where *TaHMA*s were found to be distributed on the entire set of 21 chromosomes and the additional chromosome Un [4]. A similar kind of analysis reported in the previous studies describes the distribution of 20 *GmHMA* genes on 12 chromosomes and 12 *AcHMA*s on 6 chromosomes, while also exhibiting a certain degree of propensity in the distribution of *HMA* genes on specific chromosomes as described by Fang et al. [15] and Khan et al. [5], respectively. The comparison of various physicochemical properties reveals that TaHMA1-27 have molecular weights in the range of 107.491 kDa to 42.548 kDa and pIs in the range of 5.23 to 7.23 [4,16]. Nevertheless, a broad range of values obtained for the aforementioned characteristic parameters in the present study is explainable considering that the earlier reports have worked with a smaller number of genes. Similarly, a comprehensive documentation of the molecular weights (73.11–118.12 kDa) and isoelectric points (5.0–7.8) for the 21 barley HMA proteins was reported by Zhang et al. [11], therefore emphasizing the significance of these molecular features while characterizing a gene family genome widely. Furthermore, speaking of the gene structure, a variable number of exons/introns ranging from 6 to 18 have been reported for wheat *HMA*s reported previously [4]. For *O. sativa*, *S. bicolor*, and *Z. mays*, the highest number of exons recorded is 17 each [12]. Similarly, two of the *AtHMA* genes, including *AtHMA6.1* and *AtHMA6.2*, and a *P. trichocarpa HMA* gene, i.e., *PtHMA8*, harbors the highest number of 17 exons, as reported by Zahra et al. [16] and Li et al. [14], respectively. These results are in agreement with the current study, thus suggesting the possibility of evolutionary conservedness amongst the *HMA* genes across these distinct plant species.

*Cis*-element analysis is deemed to be a crucial investigation for enlisting the promoter elements in order to predict the distinct biological roles in which HMAs are involved. A variety of *cis*-acting elements discovered for all of the *TaHMA* genes in the present investigation highlighted their putative functions in diverse roles and pathways, ranging across the regulation of growth and development, hormone signaling, regulation of abiotic factors, including light, and mediation in stress responses. Similar kinds of studies have been reported for *AtHMP*s and *OsHMP*s, where several kinds of *cis*-elements have been reported to perform an array of functions, including pollen biosynthesis and cold- and dehydration-responsive elements [8]. The results for the domain analysis obtained in the current study are in accordance with the previous reports, where there have been instances when certain characteristic HMA domains do not occur in all of the TaHMAs [4]. Similar kinds of anomalous results, where certain proteins, although belonging to the HMA superfamily, are devoid of HMA domains in Arabidopsis and rice, which was reported by Li et al. [8]. Nonetheless, the results of domain composition validated the identity of the HMA proteins. The analysis of sub-cellular localization is another widely explored parameter for any particular protein family and has been extensively deciphered for the HMAs reported in numerous plant species. For instance, the sub-cellular localization of the HvHMA proteins is predicted in the plasma membrane and the endoplasmic reticulum, while the GmHMA proteins are putatively localized in the mitochondria, the chloroplast, or are involved in the secretory pathway [11,15]. Zhiguo et al. [12] suggested variable sub-cellular localization patterns for the OsHMA, ZmHMA, and SbHMA proteins, including the chloroplast, the plasma membrane, and the tonoplast. A variable sub-cellular localization for all of the TaHMA1-27 members was also reported by Zahra et al. [16] and Batool et al. [4], the results of which support the findings in the present study, thereby consolidating the fact that the HMA proteins in *T. aestivum* are distinctly distributed across different sub-cellular components. Furthermore, the available reports on the HMA proteins have adopted variable methods to group the phylogenetic tree to ease the interpretation of the association amongst the various HMA proteins identified in any particular study [4,8,11,13,16]. Furthermore, it is well established that the HMAs belong to two prominent subfamilies, i.e., the Cu(II) and Ag(II) ATPase subfamily and the Zn(II), Pb(II), and Cd(II) ATPase subfamily [47]. Consequently, various genome-wide studies conducted for the exploration of heavy metal ATPases in different plant species, including those conducted by Batool et al. [4] and Khan et al. [5], have adopted a similar approach for the classification of HMAs. However, this method of categorizing HMAs reported in the present study based on their substrate specificity jeopardized the grouping obtained in the phylogenetic tree constructed using solely the TaHMA protein sequences. As a result, the clade-wise distribution for TaHMAs was examined based on the distribution of characteristic domains in amino acid sequences. A similar type of classification of the rice and Arabidopsis HMP proteins was done by Li et al. [8]. Moreover, the phylogenetic relationship was analyzed for the entire set of TaHMA proteins identified with the orthologous protein sequences of rice and Arabidopsis HMP proteins. Evidently, interesting inferences could be drawn with the results of phylogenetic clustering obtained in the present investigation with those of earlier reports. For instance, the member of two HMA domains containing the H4 clade, i.e., OsHMP25 was clustered together with TaHMA16-A and B, which were also seen to harbor the HMA domain twice [8]. Moreover, OsHMP09 was grouped together with TaHMA41-A, B, and D, while OsHMP19 and OsHMP39 exhibited close-knit clustering with TaHMA52 and TaHMA57 group proteins, as is evident in Figure S3. By comparing the amino acid length for TaHMA41, and TaHMA52 and TaHMA57 group proteins, it can be concluded that the sequence for the former is greater than the latter two group of proteins, and this can be well elucidated with the observation that the clades formed upon the phylogenetic association of HMA proteins differ in regard to the amino acid sequence length, as suggested by Li et al. [8]. The motif distribution analysis conducted in previously published reports on the HMA proteins also confirmed the predominance of certain characteristic motifs as observed in the present study [4,16]. A diverse and complex array of *TaHMA* genes mined using the allohexaploid genome of *T. aestivum* suggests the crucial role played by gene and genome duplication events in the evolution of the *HMA* gene family in bread wheat, similar to other plant species, such as tomato, maize, and sorghum, in which gene duplication events have also been implicated to play a key role in the expansion of the HMA family [10,12]. Moreover, the Ka/Ks ratio values reveal the proportion of beneficial as well as neutral mutations, thereby indicating whether the duplicated genes underwent positive or negative selection. As a rule of thumb, a Ka/Ks ratio value greater than one highlights positive selection, less than one indicates purifying/negative selection, while a value equal to one indicates neutral selection. Consequently, speaking of the gene duplication events analyzed in the current study, the majority of the *TaHMA* gene pairs had a Ka/Ks ratio of less than one, highlighting the significant extent to which the genes analyzed in the present study underwent purifying selection. A similar kind of trend in the values for the aforementioned parameter is documented for the duplicated *GmHMA* (Ka/Ks = 0.05–0.66) and *SlHMA* (Ka/Ks = 0.17–0.38) gene pairs reported in soybean and tomato, respectively, thereby unequivocally confirming the plausibility of a similar kind of selection occurring in the *HMA* genes of bread wheat [10,15]. The results obtained for the synteny analysis conducted in order to decipher the evolutionary linkage using the amino acid sequences of AtHMAs, OsHMAs, and TaHMAs were in accordance with those obtained through the phylogenetic analysis, thereby strengthening the validity of the results obtained through two entirely different tools and algorithms employed in the present study. Moreover, syntenic analysis carried out on similar lines has been reported for the *TaHMA* genes recognized previously, where they prominently stood out for having been evolutionarily related to the *AtHMA* genes [16].

The analysis of expression constitutes a critical component for the characterization of any particular gene family under study since it aids in deciphering the role of gene-encoding proteins in the vital biological processes of a living organism, as in this case for bread wheat. The highly expressed *TaHMA* genes in the root tissue, i.e., *TaHMA90* and *TaHMA85* group genes, were orthologs with *OsHMP28*, which exhibits root-specific expression, and *AtHMP29*, exhibiting a high expression level in the fruit of Arabidopsis and was related to *TaHMA58* group genes, which were exclusively expressed in the later developmental stages of the grain in bread wheat (Figure 8 and Figure S3). Furthermore, various lowly expressed genes, such as *TaHMA44-A*, *B*, and *D*, *TaHMA52-B* and *D*, *TaHMA1-A*, *B*, and *D*, *TaHMA7-A*, *B*, and *D*, and *TaHMA75-D* were phylogenetically clustered close to *OsHMP6*, *OsHMP16*, *OsHMP30*, *OsHMP31*, and *OsHMP41*, all of which also have a minimal expression profile in various tissues, including the carpel, leaves, pistil, and embryo [8]. *TaHMA90* group genes showing consistently high expression across various developmental stages of vegetative organs, including root, stem, and leaf, were phylogenetically related to *AtHMP14* and *AtHMP31*, both of which also had high transcript levels in the majority of the tissues examined by Li et al. [8]. Moreover, comparing the expression pattern with another evident parameter, such as the sub-cellular localization, also revealed interesting results. For instance, OsHMA5 (OsHMP27) and OsHMA9 (OsHMP33) are reported to be localized in the cell membrane and are involved in mediating the transport of heavy metals in the root and shoot [4,8]. Its orthologous counterparts, i.e., *TaHMA91* (*-B* and *D*) and *TaHMA23* (*-A* and *B*), respectively, as evident from the phylogenetic tree (Figure S3), also exhibited some significant expression in the root and were predicted to be localised in the plasma membrane, thereby establishing a similar role for the HMAs under study (Figure 8). A similar kind of differential results have been obtained for the expression of *SlHMA* genes in various tissue types, including roots, leaves, and flower buds of tomato [10]. Additionally, the occurrence of various stress-responsive *cis*-regulatory elements in the promoter region of the *TaHMA* genes, such as ABA-responsive and dehydration-responsive elements (ABRE and DRE core), validated the drought and salinity stress-mediated differential gene expression fold-change observed for the *TaHMA*s in the present study [48]. Furthermore, *cis*-element analysis conducted for all of the *AtHMP*s and *OsHMP*s by Li et al. [8] also revealed the presence of ABA as well as cold- and dehydration-responsive elements, thereby elucidating the role of these heavy metal-associated proteins encoding genes involved in the regulation of stress response and strengthening the findings observed for the differentially expressed *TaHMA* genes under stress in the present study. Therefore, a combination of available scientific literature along with the collective evidence from the present study potently highlights the noticeable role of *HMA* genes in plant growth, development, and stress responses.

## 5. Conclusions

This study successfully reported the full complement of the *TaHMA* gene family, consisting of 243 members in bread wheat. The results of chromosomal distribution revealed that *HMA* genes occurred on the entire set of monoploid chromosomes in wheat, followed by a variable intron/exon number, which is in agreement with previous studies, thus suggesting a plausible evolutionary relatedness with various plant species. Diverse cellular compartmentalization, followed by conserved domain architecture and a clade-wise distribution of the TaHMAs, offered fruitful insights and is in agreement with previous studies conducted for model plant species. A variety of *cis*-regulatory elements discovered in the promoter region of *TaHMA* genes, particularly those participating in stress responsiveness, elucidated the function of HMAs in alleviating the effect of any resistance or stress encountered by the plant body. The effect of a few such factors, which were examined in the present study, including heat, drought, and salt stress through the RNA-seq data, further validated the stress-specific role of *TaHMA*s and also positioned them to play a significant role in plant growth and development. Overall, this study widened the gene pool of heavy metal ATPases reported in bread wheat followed by their comprehensive characterization and expression profiling, which created a robust foundation for future functional annotation studies.

## Data Availability

All data are available in the manuscript and Supplementary Files.

## Supplementary Materials

The following supporting information can be downloaded at: https://www.mdpi.com/article/10.3390/biology14070818/s1, Figure S1: Sub-cellular localization signal values obtained using the WoLF PSORT tool for the 243 TaHMA proteins identified in the present study. The signal values were plotted in a way that the homoeologous TaHMAs were grouped together in a single plot. In certain cases, where the homoeologous counterparts were non-occurring, the neighboring IDs with consecutive nomenclature were grouped together to ease the presentation of data; Figure S2: Gene-wise distribution of the cis-regulatory elements (growth and development specific, light responsive, hormone responsive, and stress responsive) generated using TBtools; Figure S3: Unrooted phylogenetic tree for the 46, 55, and 243 full-length amino acid sequences of Arabidopsis and rice HMP proteins [8] and TaHMA proteins (present study), respectively, generated by MEGA7 and refined using iTOL v7; Figure S4: Motif distribution for the 243 TaHMA proteins retrieved using MEME Suite 5.5.7 and the resultant graphic diagrams generated using TBtools in a sub-genome-wise manner to ease visualization; Table S1: Physicochemical characteristics of TaHMA genes and their encoded proteins in *Triticum aestivum*; Table S2: List of duplicated TaHMA gene pairs with their Ka/Ks ratios and estimated divergence time (MYA).
