# Supplementary material for: Heavy Metal-Associated (HMA) Domain-Containing Proteins: Insight into Their Features and Roles in Bread Wheat (Triticum aestivum L.)
Source: Biology (Basel). 2025 Jul 5;14(7):818. doi: 10.3390/biology14070818 (PMC12292569; doi:10.3390/biology14070818)
Supplement: Supplementary file 1 [file biology-14-00818-s001.zip › Supplementary_figure S1.pdf]

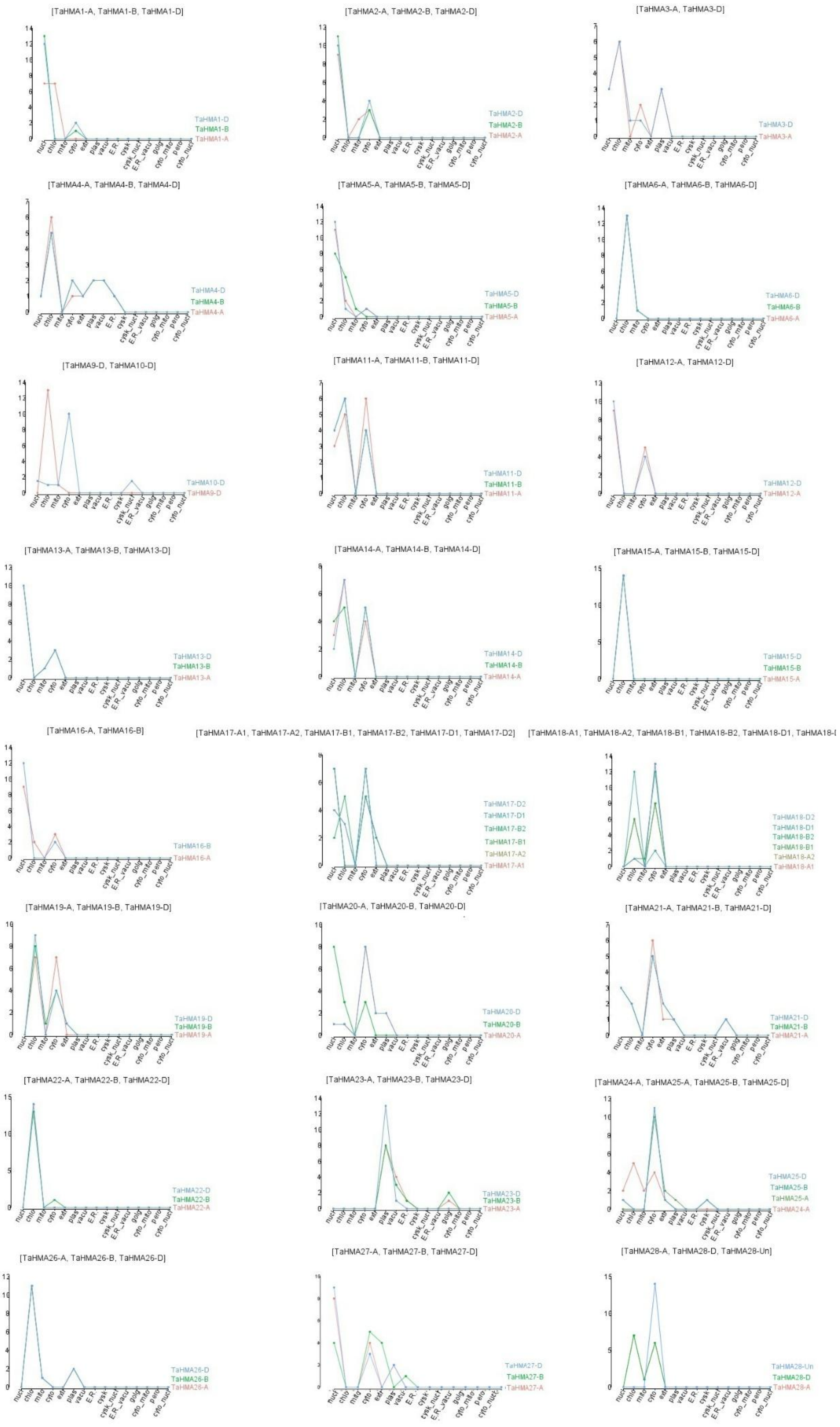
[TaHMA1-A, TaHMA1-B, TaHMA1-D]
TaHMA1-D
TaHMA1-B
TaHMA1-A
[TaHMA2-A, TaHMA2-B, TaHMA2-D]
TaHMA2-D
TaHMA2-B
TaHMA2-A
[TaHMA3-A, TaHMA3-D]
TaHMA3-D
TaHMA3-A
[TaHMA4-A, TaHMA4-B, TaHMA4-D]
TaHMA4-D
TaHMA4-B
TaHMA4-A
[TaHMA5-A, TaHMA5-B, TaHMA5-D]
TaHMA5-D
TaHMA5-B
TaHMA5-A
[TaHMA6-A, TaHMA6-B, TaHMA6-D]
TaHMA6-D
TaHMA6-B
TaHMA6-A
[TaHMA9-D, TaHMA10-D]
TaHMA10-D
TaHMA9-D
[TaHMA11-A, TaHMA11-B, TaHMA11-D]
TaHMA11-D
TaHMA11-B
TaHMA11-A
[TaHMA12-A, TaHMA12-D]
TaHMA12-D
TaHMA12-A
[TaHMA13-A, TaHMA13-B, TaHMA13-D]
TaHMA13-D
TaHMA13-B
TaHMA13-A
[TaHMA14-A, TaHMA14-B, TaHMA14-D]
TaHMA14-D
TaHMA14-B
TaHMA14-A
[TaHMA15-A, TaHMA15-B, TaHMA15-D]
TaHMA15-D
TaHMA15-B
TaHMA15-A
[TaHMA16-A, TaHMA16-B]
TaHMA16-B
TaHMA16-A
[TaHMA17-A1, TaHMA17-A2, TaHMA17-B1, TaHMA17-B2, TaHMA17-D1, TaHMA17-D2]
TaHMA17-D2
TaHMA17-D1
TaHMA17-B2
TaHMA17-B1
TaHMA17-A2
TaHMA17-A1
[TaHMA18-A1, TaHMA18-A2, TaHMA18-B1, TaHMA18-B2, TaHMA18-D1, TaHMA18-
TaHMA18-D2
TaHMA18-D1
TaHMA18-B2
TaHMA18-B1
TaHMA18-A2
TaHMA18-A1
[TaHMA19-A, TaHMA19-B, TaHMA19-D]
TaHMA19-D
TaHMA19-B
TaHMA19-A
[TaHMA20-A, TaHMA20-B, TaHMA20-D]
TaHMA20-D
TaHMA20-B
TaHMA20-A
[TaHMA21-A, TaHMA21-B, TaHMA21-D]
TaHMA21-D
TaHMA21-B
TaHMA21-A
[TaHMA22-A, TaHMA22-B, TaHMA22-D]
TaHMA22-D
TaHMA22-B
TaHMA22-A
[TaHMA23-A, TaHMA23-B, TaHMA23-D]
TaHMA23-D
TaHMA23-B
TaHMA23-A
[TaHMA24-A, TaHMA25-A, TaHMA25-B, TaHMA25-D]
TaHMA25-D
TaHMA25-B
TaHMA25-A
TaHMA24-A
[TaHMA26-A, TaHMA26-B, TaHMA26-D]
TaHMA26-D
TaHMA26-B
TaHMA26-A
[TaHMA27-A, TaHMA27-B, TaHMA27-D]
TaHMA27-D
TaHMA27-B
TaHMA27-A
[TaHMA28-A, TaHMA28-D, TaHMA28-Un]
TaHMA28-Un
TaHMA28-D
TaHMA28-A
nucl chlo mito cyto extr plas vacu E.R. cysk cysk_nucl E.R._vacu golg cyto_mito pero cyto_nucl

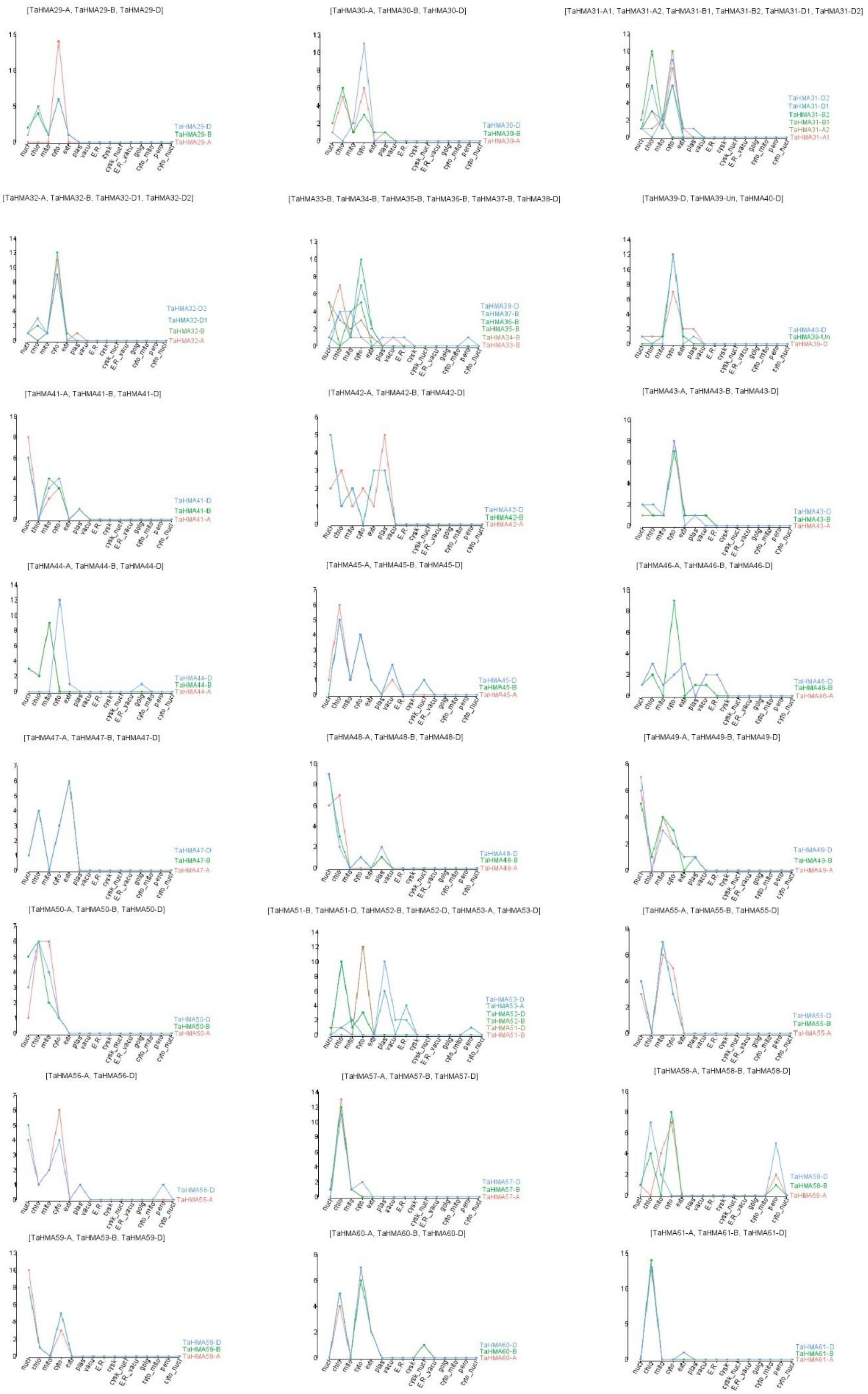
[TaHMA29-A, TaHMA29-B, TaHMA29-D]
TaHMA29-D
TaHMA29-B
TaHMA29-A
[TaHMA30-A, TaHMA30-B, TaHMA30-D]
TaHMA30-D
TaHMA30-B
TaHMA30-A
[TaHMA31-A1, TaHMA31-A2, TaHMA31-B1, TaHMA31-B2, TaHMA31-D1, TaHMA31-D2]
TaHMA31-D2
TaHMA31-D1
TaHMA31-B2
TaHMA31-B1
TaHMA31-A2
TaHMA31-A1
[TaHMA32-A, TaHMA32-B, TaHMA32-D1, TaHMA32-D2]
TaHMA32-D2
TaHMA32-D1
TaHMA32-B
TaHMA32-A
[TaHMA33-B, TaHMA34-B, TaHMA35-B, TaHMA36-B, TaHMA37-B, TaHMA38-D]
TaHMA38-D
TaHMA37-B
TaHMA36-B
TaHMA35-B
TaHMA34-B
TaHMA33-B
[TaHMA39-D, TaHMA39-Un, TaHMA40-D]
TaHMA40-D
TaHMA39-Un
TaHMA39-D
[TaHMA41-A, TaHMA41-B, TaHMA41-D]
TaHMA41-D
TaHMA41-B
TaHMA41-A
[TaHMA42-A, TaHMA42-B, TaHMA42-D]
TaHMA42-D
TaHMA42-B
TaHMA42-A
[TaHMA43-A, TaHMA43-B, TaHMA43-D]
TaHMA43-D
TaHMA43-B
TaHMA43-A
[TaHMA44-A, TaHMA44-B, TaHMA44-D]
TaHMA44-D
TaHMA44-B
TaHMA44-A
[TaHMA45-A, TaHMA45-B, TaHMA45-D]
TaHMA45-D
TaHMA45-B
TaHMA45-A
[TaHMA46-A, TaHMA46-B, TaHMA46-D]
TaHMA46-D
TaHMA46-B
TaHMA46-A
[TaHMA47-A, TaHMA47-B, TaHMA47-D]
TaHMA47-D
TaHMA47-B
TaHMA47-A
[TaHMA48-A, TaHMA48-B, TaHMA48-D]
TaHMA48-D
TaHMA48-B
TaHMA48-A
[TaHMA49-A, TaHMA49-B, TaHMA49-D]
TaHMA49-D
TaHMA49-B
TaHMA49-A
[TaHMA50-A, TaHMA50-B, TaHMA50-D]
TaHMA50-D
TaHMA50-B
TaHMA50-A
[TaHMA51-B, TaHMA51-D, TaHMA52-B, TaHMA52-D, TaHMA53-A, TaHMA53-D]
TaHMA53-D
TaHMA53-A
TaHMA52-D
TaHMA52-B
TaHMA51-D
TaHMA51-B
[TaHMA55-A, TaHMA55-B, TaHMA55-D]
TaHMA55-D
TaHMA55-B
TaHMA55-A
[TaHMA56-A, TaHMA56-D]
TaHMA56-D
TaHMA56-A
[TaHMA57-A, TaHMA57-B, TaHMA57-D]
TaHMA57-D
TaHMA57-B
TaHMA57-A
[TaHMA58-A, TaHMA58-B, TaHMA58-D]
TaHMA58-D
TaHMA58-B
TaHMA58-A
[TaHMA59-A, TaHMA59-B, TaHMA59-D]
TaHMA59-D
TaHMA59-B
TaHMA59-A
[TaHMA60-A, TaHMA60-B, TaHMA60-D]
TaHMA60-D
TaHMA60-B
TaHMA60-A
[TaHMA61-A, TaHMA61-B, TaHMA61-D]
TaHMA61-D
TaHMA61-B
TaHMA61-A
nucl chlo mito cyto extr plas vacu E.R. cysk cysk_nucl E.R._vacu golg cyto_mito pero cyto_nucl

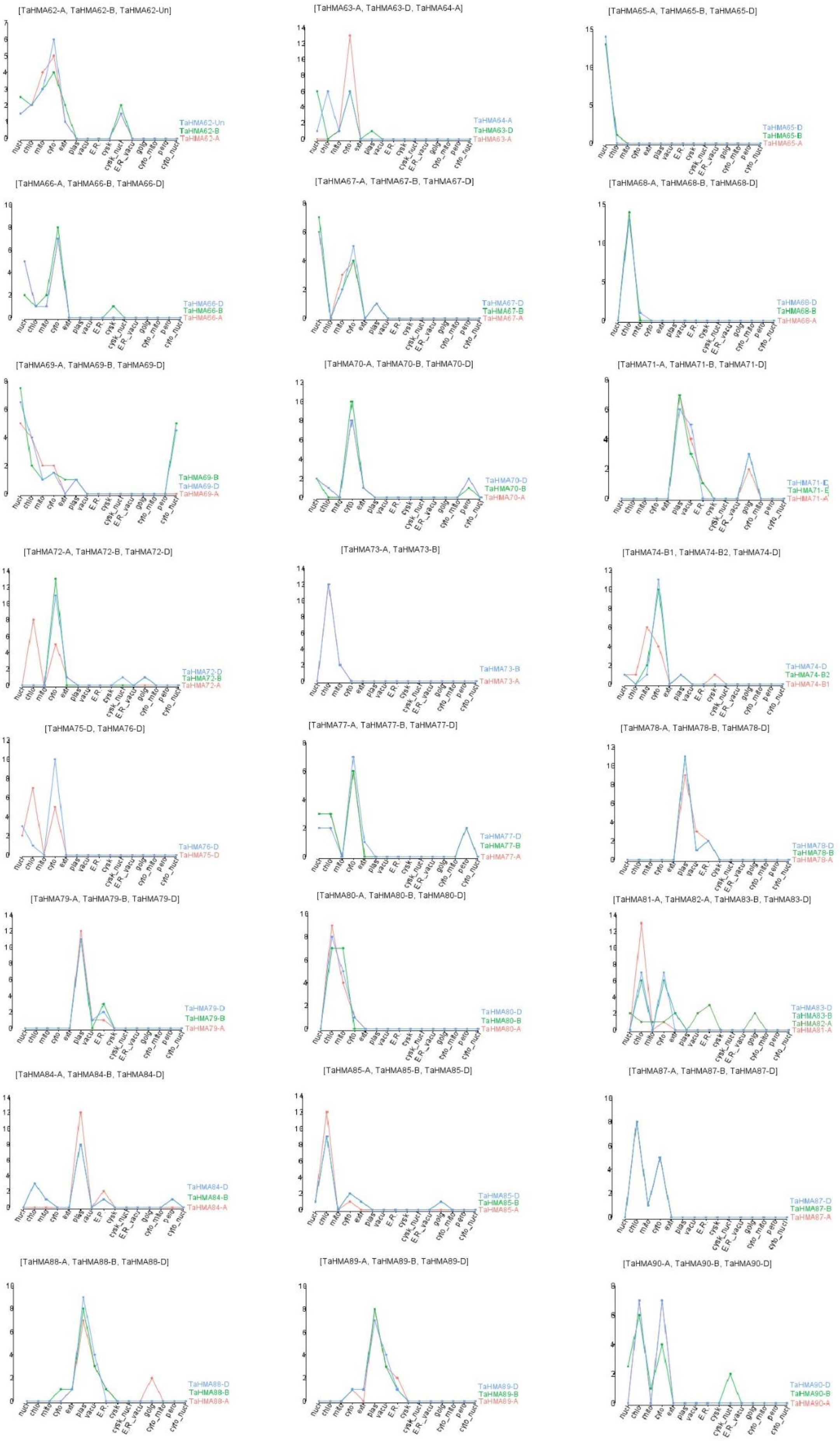

[TaHMA62-A, TaHMA62-B, TaHMA62-Un]
TaHMA62-Un
TaHMA62-B
TaHMA62-A
[TaHMA63-A, TaHMA63-D, TaHMA64-A]
TaHMA64-A
TaHMA63-D
TaHMA63-A
[TaHMA65-A, TaHMA65-B, TaHMA65-D]
TaHMA65-D
TaHMA65-B
TaHMA65-A
[TaHMA66-A, TaHMA66-B, TaHMA66-D]
TaHMA66-D
TaHMA66-B
TaHMA66-A
[TaHMA67-A, TaHMA67-B, TaHMA67-D]
TaHMA67-D
TaHMA67-B
TaHMA67-A
[TaHMA68-A, TaHMA68-B, TaHMA68-D]
TaHMA68-D
TaHMA68-B
TaHMA68-A
[TaHMA69-A, TaHMA69-B, TaHMA69-D]
TaHMA69-B
TaHMA69-D
TaHMA69-A
[TaHMA70-A, TaHMA70-B, TaHMA70-D]
TaHMA70-D
TaHMA70-B
TaHMA70-A
[TaHMA71-A, TaHMA71-B, TaHMA71-D]
[TaHMA72-A, TaHMA72-B, TaHMA72-D]
TaHMA72-D
TaHMA72-B
TaHMA72-A
[TaHMA73-A, TaHMA73-B]
TaHMA73-B
TaHMA73-A
[TaHMA74-B1, TaHMA74-B2, TaHMA74-D]
TaHMA74-D
TaHMA74-B2
TaHMA74-B1
[TaHMA75-D, TaHMA76-D]
TaHMA76-D
TaHMA75-D
[TaHMA77-A, TaHMA77-B, TaHMA77-D]
TaHMA77-D
TaHMA77-B
TaHMA77-A
[TaHMA78-A, TaHMA78-B, TaHMA78-D]
TaHMA78-D
TaHMA78-B
TaHMA78-A
[TaHMA79-A, TaHMA79-B, TaHMA79-D]
TaHMA79-D
TaHMA79-B
TaHMA79-A
[TaHMA80-A, TaHMA80-B, TaHMA80-D]
TaHMA80-D
TaHMA80-B
TaHMA80-A
[TaHMA81-A, TaHMA82-A, TaHMA83-B, TaHMA83-D]
TaHMA83-D
TaHMA83-B
TaHMA82-A
TaHMA81-A
[TaHMA84-A, TaHMA84-B, TaHMA84-D]
TaHMA84-D
TaHMA84-B
TaHMA84-A
[TaHMA85-A, TaHMA85-B, TaHMA85-D]
TaHMA85-D
TaHMA85-B
TaHMA85-A
[TaHMA87-A, TaHMA87-B, TaHMA87-D]
TaHMA87-D
TaHMA87-B
TaHMA87-A
[TaHMA88-A, TaHMA88-B, TaHMA88-D]
TaHMA88-D
TaHMA88-B
TaHMA88-A
[TaHMA89-A, TaHMA89-B, TaHMA89-D]
TaHMA89-D
TaHMA89-B
TaHMA89-A
[TaHMA90-A, TaHMA90-B, TaHMA90-D]
TaHMA90-D
TaHMA90-B
TaHMA90-A
nucl
chlo
mito
cyto
extr
plas
vacu
E.R.
cysk
cysk_nucl
E.R._vacu
golg
cyto_mito
pero
cyto_nucl

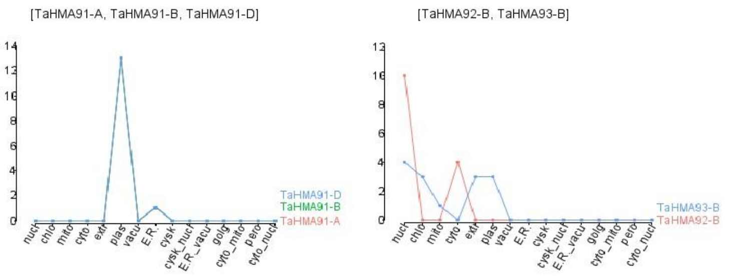

[TaHMA91-A, TaHMA91-B, TaHMA91-D]
TaHMA91-D
TaHMA91-B
TaHMA91-A
nucl
chlo
mito
cyto
extr
plas
vacu
E.R.
cysk
cysk_nucl
E.R._vacu
golg
cyto_mito
pero
cyto_nucl
[TaHMA92-B, TaHMA93-B]
TaHMA93-B
TaHMA92-B
