# Supplementary material for: Heavy Metal-Associated (HMA) Domain-Containing Proteins: Insight into Their Features and Roles in Bread Wheat (Triticum aestivum L.)
Source: Biology (Basel). 2025 Jul 5;14(7):818. doi: 10.3390/biology14070818 (PMC12292569; doi:10.3390/biology14070818)
Supplement: Supplementary file 1 [file biology-14-00818-s001.zip › Supplementary_figure S2.pdf]

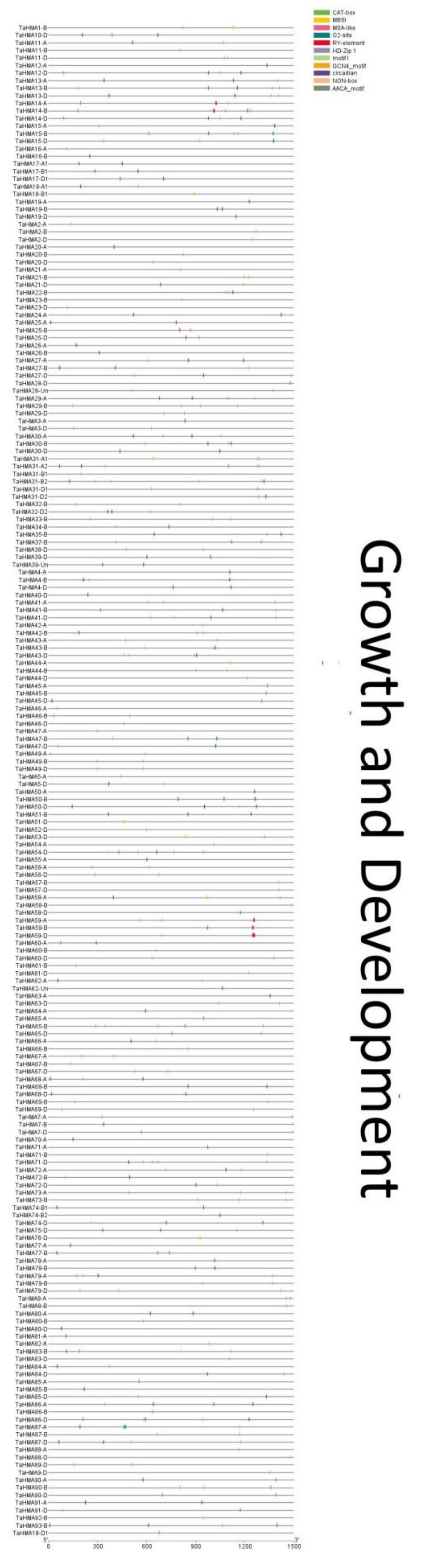

CAT-box
MBSI
MSA-like
O2-site
RY-element
HD-Zip 1
motif I
GCN4_motif
circadian
NON-box
AACA_motif
Growth and Development
5'
3'
0
300
600
900
1200
1500

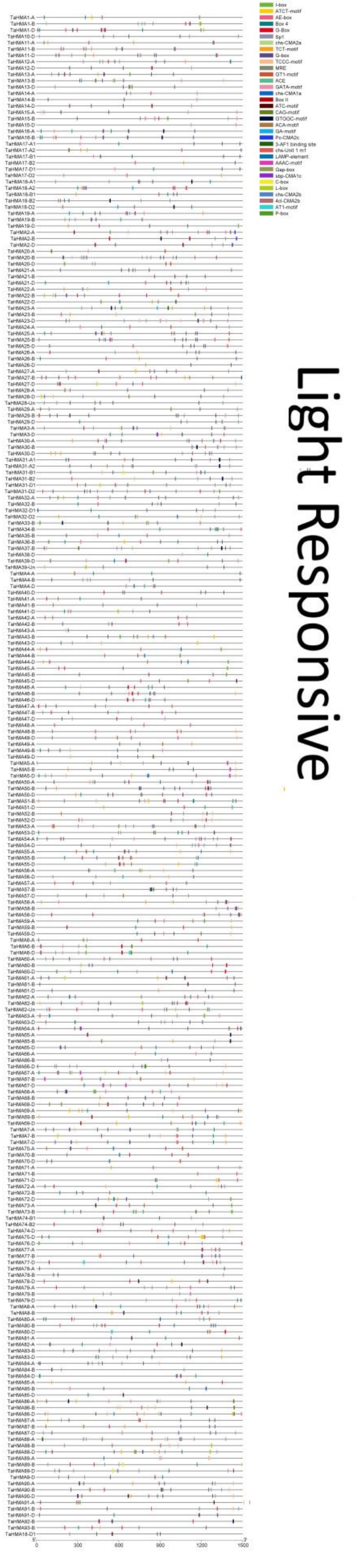
I-box
ATCT-motif
AE-box
Box 4
G-Box
Sp1
chs-CMA2a
TCT-motif
G-box
TCCC-motif
MRE
GT1-motif
ACE
GATA-motif
chs-CMA1a
Box II
ATC-motif
CAG-motif
GTGGC-motif
ACA-motif
GA-motif
Pc-CMA2c
3-AF1 binding site
chs-Unit 1 m1
LAMP-element
AAAC-motif
Gap-box
sbp-CMA1c
C-box
L-box
chs-CMA2b
4cl-CMA2b
AT1-motif
P-box
5'
3'
0
300
600
900
1200
1500
Light Responsive

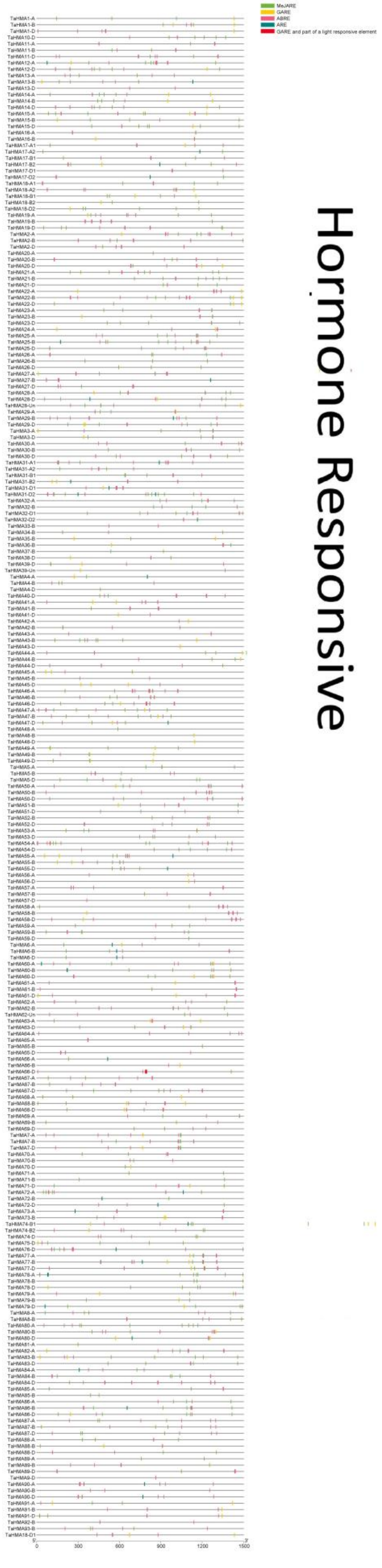
MeJARE
GARE
ABRE
ARE
GARE and part of a light responsive element
Hormone Responsive
5'
3'
0
300
600
900
1200
1500

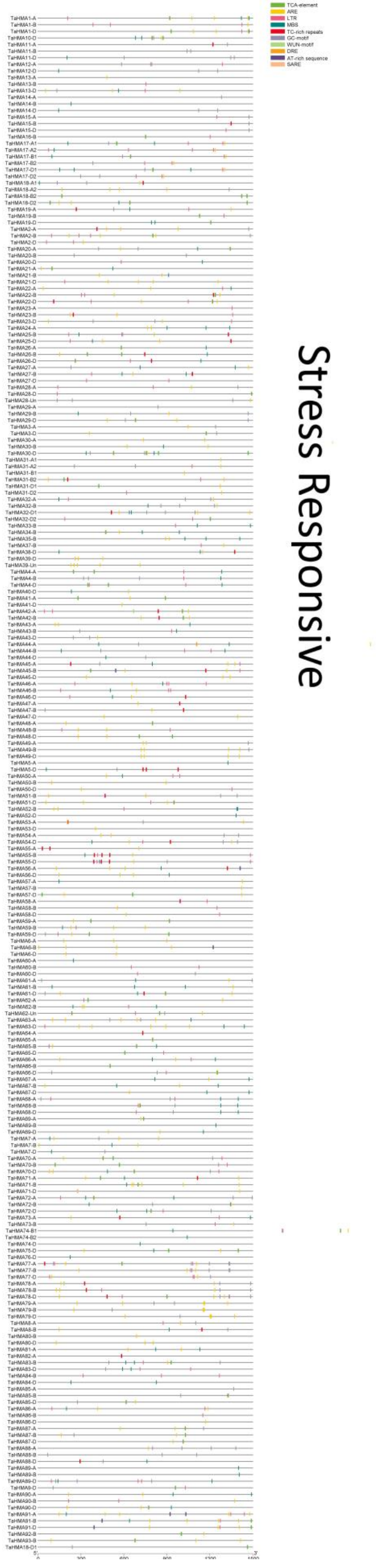
TCA-element
ARE
LTR
MBS
TC-rich repeats
GC-motif
WUN-motif
DRE
AT-rich sequence
SARE
Stress Responsive
5'
3'
0
300
600
900
1200
1500
