# Supplementary figures and images for: Heavy Metal-Associated (HMA) Domain-Containing Proteins: Insight into Their Features and Roles in Bread Wheat (Triticum aestivum L.)
Source: Biology (Basel). 2025 Jul 5;14(7):818. doi: 10.3390/biology14070818 (PMC12292569; doi:10.3390/biology14070818)

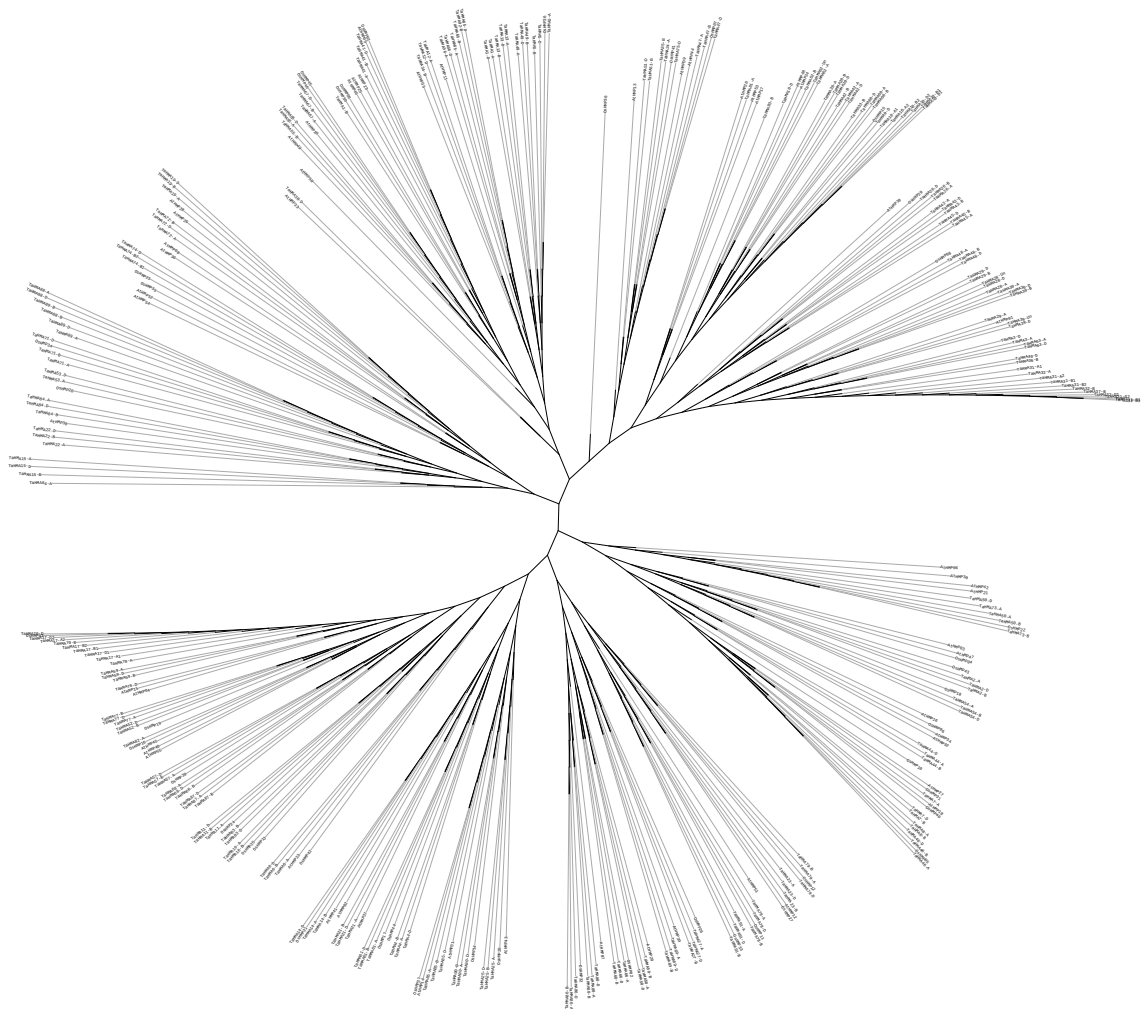

Supplement: Supplementary file 1 [file biology-14-00818-s001.zip › Supplementary_figure S3.pdf]
