# Supplementary material for: Heavy Metal-Associated (HMA) Domain-Containing Proteins: Insight into Their Features and Roles in Bread Wheat (Triticum aestivum L.)
Source: Biology (Basel). 2025 Jul 5;14(7):818. doi: 10.3390/biology14070818 (PMC12292569; doi:10.3390/biology14070818)
Supplement: Supplementary file 1 [file Supplementary_figureS4.pdf › Supplementary_figureS4.pdf]

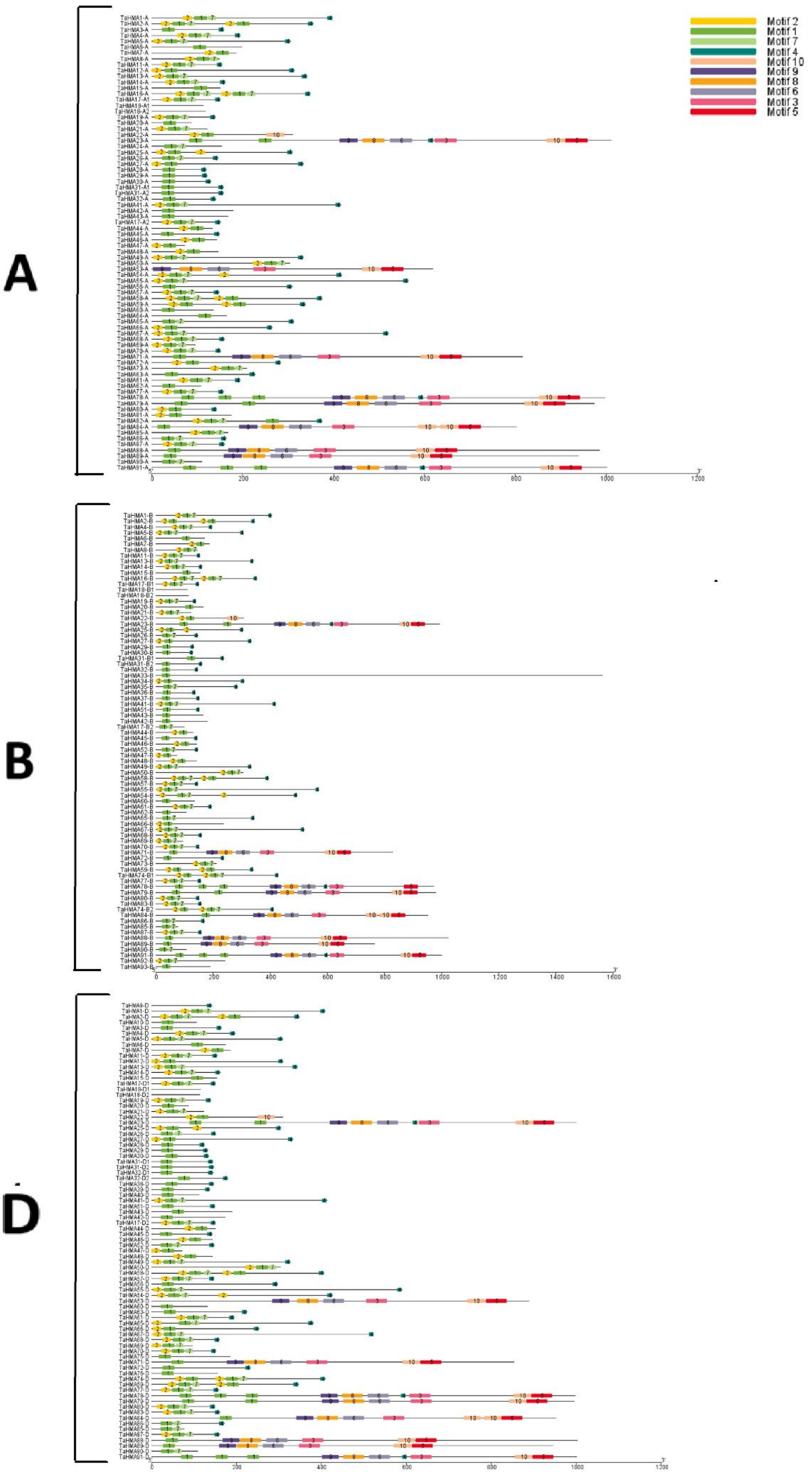
A
B
D
Motif 2
Motif 1
Motif 7
Motif 4
Motif 10
Motif 9
Motif 8
Motif 6
Motif 3
Motif 5
